# Alpelisib-Induced Diabetes Mellitus: Case Report, Pharmacodynamics and Management Considerations

**DOI:** 10.3389/fendo.2022.802612

**Published:** 2022-01-31

**Authors:** Begoña Pla Peris, Alfonso Arranz Martin, Anabel Ballesteros García, Fernando Sebastián-Valles, Monica Marazuela Azpiroz

**Affiliations:** ^1^ Department of Endocrinology and Nutrition, Hospital Universitario de Castellón, Castellón, Spain; ^2^ Department of Endocrinology and Nutrition, Instituto de Investigación Princesa, Universidad Autónoma de Madrid, Hospital Universitario de La Princesa, Madrid, Spain; ^3^ Department of Oncology, Hospital Universitario de La Princesa, Madrid, Spain

**Keywords:** PI3K inhibitor, alpelisib, hyperglycemia, diabetes mellitus, flash glucose monitoring (FGM)

## Abstract

**Introduction:**

Alpelisib is an orally selective PI3K alpha inhibitor recently available for the treatment of advanced breast cancer. PI3K pathway is an intracellular signaling pathway that plays an important role in regulating glucose metabolism. Hyperglycemia is the most common adverse event associated.

**Methods:**

We describe the case of a severe hyperglycemia associated with alpelisib treatment in a patient with metastatic breast cancer and previously near-normal glycemia. We analyze the clinical presentation, PI3K inhibitor pharmacodynamic aspects, its influence in glycemic control and the required treatment approach.

**Results:**

An important impairment of glycemic control was observed after initiation of alpelisib. In addition to insulin sensitizers drugs, intensive insulin regimen was necessary. Flash glucose monitoring (FGM) information has been helpful in understanding the pharmacodynamic aspects of alpelisib and insulin titration. Development of hyperglycemia is fast, already observed 24 hours after initiation of therapy. FGM shows severe and persistent hyperglycemia during most of the day, with a significant downward effect in the 4 hours after each daily intake, which evidences the strong but transitory effect of the drug enzyme blockade. C-peptide level is remarkable in accordance with drug pharmacodynamics, consistent with a significant insulin resistance.

**Conclusions:**

Glucose monitoring should always be performed in patients treated with alpelisib, especially in patients with diabetes and prediabetes. It is crucial to anticipate in these patients. Any delay can lead to a worsening in metabolic control resulting in the discontinuation or reduction of alpelisib, which would lead to a decrease in its effectiveness, and consequently would deny patients an effective treatment with an impact on survival.

## Introduction

Breast cancer is the most common cancer worldwide with more than 2.2 million cases in 2020. There is 1 in 12 chance women will develop breast cancer sometime in their lives (1).

Breast cancer can be divided into 3 major biological subgroups, each of which directly influences on the treatment choice: estrogen receptor (ER)-positive, human epidermal growth factor receptor 2 (HER2)-positive breast cancer and ER/HER2 and progesterone (PR)-negative breast cancer (triple negative). More than 70% of breast cancers are HR-positive and HER2-negative, and 40% of them have activating mutations in the phosphatidylinosiyol 3-kinase (PI3K) gene (2).

Alpelisib is an orally selective PI3K alpha inhibitor recently available for the treatment of advanced breast cancer. Alpelisib plus fulvestrant (classified as an estrogen receptor antagonist) have synergistic antitumor activity and are used in patients with PIK3CA-mutated, HR positive, HER2-negative advanced breast cancer who had received endocrine therapy previously as cyclin-dependent kinase 4 and 6 (CDK4/6) inhibitors (3).

PI3K is part of an intracellular signaling pathway that plays an important role in regulating glucose metabolism and cellular proliferation. In many cancers this pathway is frequently dysregulated, allowing oncogenesis and resistance to treatment (4). Additionally, the role of PI3K in glucose homeostasis also results in new onset hyperglycemia, which is observed with PI3K inhibition (5, 6).

Clinically relevant adverse events are expected with alpelisib treatment, and hyperglycemia in particular is the most common adverse event associated. It occurs in 59% of patients, 28% occurring in moderate (grade 3 toxicity - fasting plasma glucose > 250 to 500 mg/dl) and severe (grade 4 toxicity - fasting plasma glucose > 500 mg/dl) forms (6). Even if in clinical trials history of diabetes excludes the use of PI3K inhibitor, adverse events (mainly hyperglycemia) lead to treatment discontinuation is 20% of patients, despite closing monitoring (7).

We describe the case of a severe hyperglycemia associated with alpelisib treatment in a patient with metastatic breast cancer. We analyse the clinical presentation, PI3K inhibitor pharmacodynamic aspects, its influence in glycemic control and the required treatment approach that we believe is crucial to anticipate in these patients with an effective oncologic treatment.

## Case Description

**Figure 1:**
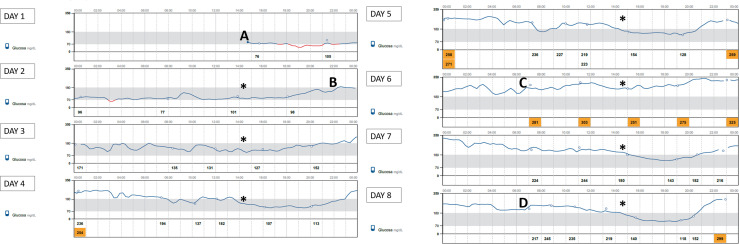
FreeStyle Libre 2^®^ Abbott Glucose Monitoring (LibreView system) following start of alpelisib **(A)** Alpelisib start (300 mg/daily); **(B)** Metformin 850 mg daily addition; **(C)** Alogliptin 25 mg and pioglitazone 30 mg daily addition; **(D)** insulin addition, *Alpelisib daily dose).

**Figure 2:**
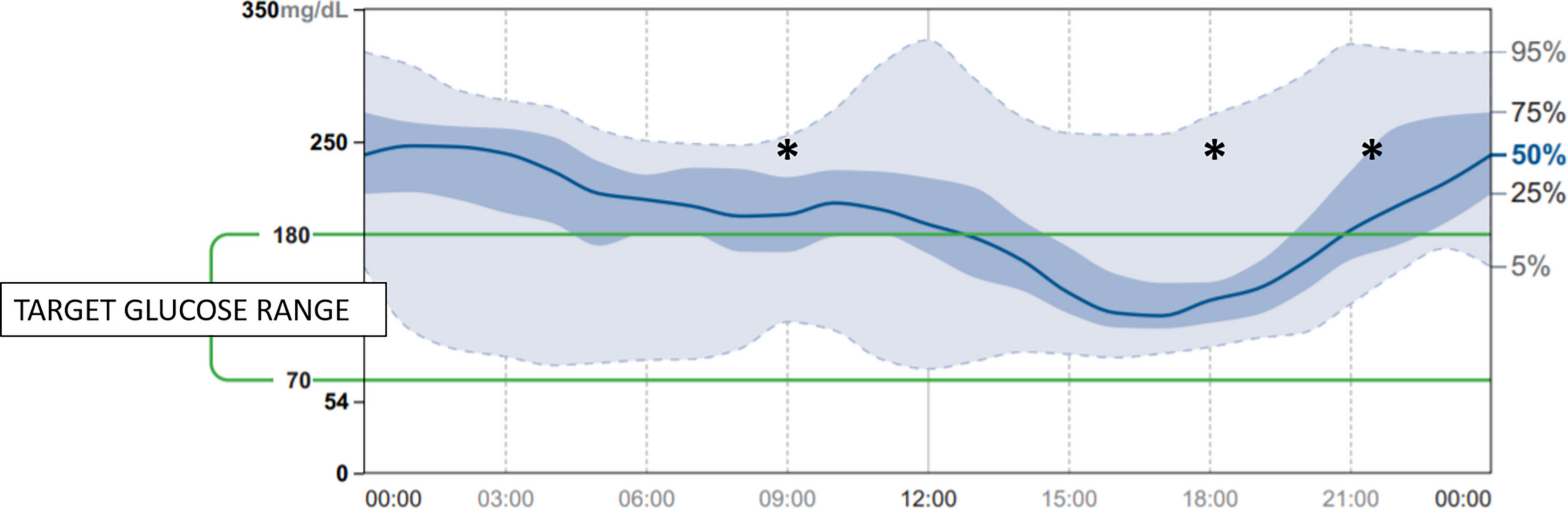
14 days Ambulatory Glucose Profile following start of alpelisib [insulin dose-titration period] (*: prandial insulin coverage with correction boluses at 0900h, 1800h, 2130h).

**Figure 3:**
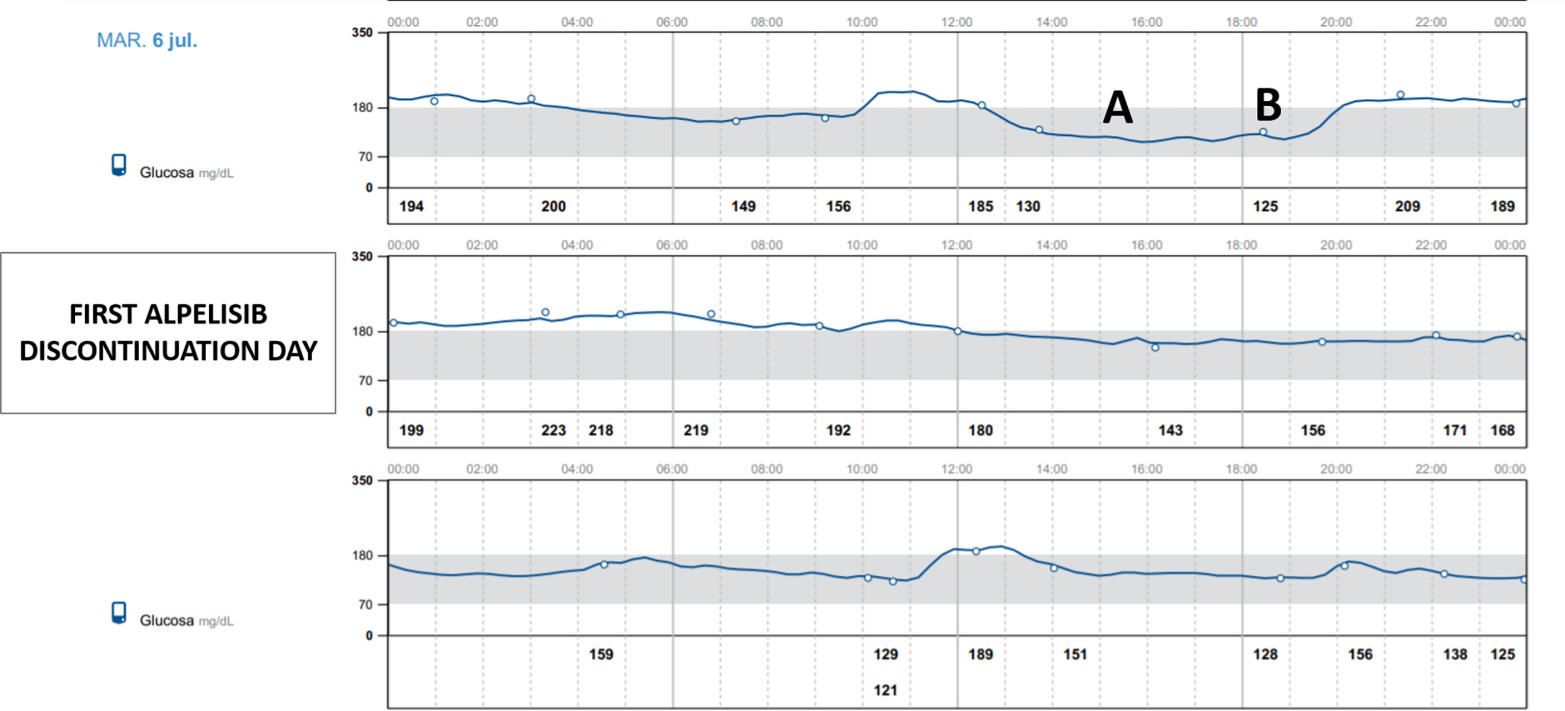
FreeStyle Libre 2^®^ Abbott Glucose Monitoring (LibreView system) following alpelisib discontinuation. **(A)** Last alpelisib daily dose, **(B)** Last insulin dose).

68-year-old female with history of bilateral infiltrating ductal carcinoma breast cancer since 2011 treated with bilateral radical mastectomy. Despite having received endocrine therapy previously and chemotherapy, laboratory findings revealed an increase in tumor markers. PI3KCA mutation was present and treatment with alpelisib was approved. She did not have a previous medical history of diabetes mellitus but 7 days before starting treatment her fasting plasma glucose was 103 mg/dl and her HbA1c was 5.8%. Her body mass index was 27.3 kg/m^2^. Given the presence of mild dysglycemia with prediabetes criteria, we prospectively used flash glucose monitoring system (FGM) FreeStyle Libre 2^®^ Abbott and a sequential therapy with antidiabetic drugs was established in case hyperglycemia rose to 160 mg/dl. Hyperglycemia was already observed on the first day of treatment with alpelisib (**Figure 1**). Close follow-up was conducted. Treatment with metformin 850 mg daily was started. Addition of alogliptin 25 mg daily and pioglitazone 30 mg daily was necessary on day 6 of treatment with alpelisib, and on day 8 concomitant insulin therapy (initial dose 14 UI/day) was needed in order to improve glycemic control that was poor despite the close follow-up and despite the sequential therapy addition with the referred antidiabetic drugs (**Figure 1**). 7 days Ambulatory Glucose Profile following start of alpelisib are representative of this period and showed: average glucose 178 mg/dl, time in range (TIR) [70-180] 50%, glucose management indicator (GMI) 7.8%, percentage of time abow 180 mg/dl 47%, percentage of time below 70 mg/dl 3%. Following FGM data, an increase in basal insulin dose and the addition of prandial insulin coverage with correction boluses were needed. **Figure 2** shows 14 days Ambulatory Glucose Profile following start of alpelisib (insulin dose-titration period). Relative glycemic stability was achieved with a total insulin daily dose of 38 UI, corresponding to 0.56 UI/kg, which included 26 UI of basal insulin and 12 units of prandial insulin (68.4% of basal insulin/31.5% of prandial insulin). 14 days Ambulatory Glucose Profile in this glycemic stability period showed: average glucose 158 mg/dl, time in range (TIR) [70-180] 71%, glucose management indicator (GMI) 7.1%, coefficient of variation (CV) 26.4%, percentage of time abow 180 mg/dl 27%, percentage of time abow 250 mg/dl 2%, percentage of time below 70 mg/dl 1%, percentage of time below 54 mg/dl 0%. GAD and IA-2 antibodies were negative (0.1U/mL; [positive ≥ 5U/mL and ≥ 15, respectively) and fasting C-peptide level was significantly high (13.9 ng/ml [0.9-3.1]) showing a strong insulin resistance. Besides hyperglycemia, alpelisib was remarkably well tolerated as well as its impact in tumor markers evolution (**Table 1**). 4 months later the patient was admitted to hospital because of fever without a focus. Plasma glycated hemoglobin (HbA1c) at month 4 was 8%. Alpelisib was interrupted and glycemia normalized in the next 24 hours (**Figure 3**) with no need of insulin continuation. PET-CT with 18 107 F- FDG showed a favorable response compared to PET-CT performed prior to the initiation of alpelisib, with persistence of tumor metabolic activity in the lytic-blastic lesions described in mediastinal lymph nodes and left costal pleura, all of which had a lower SUVmax (costal pleura nodes: SUVmax 4.3; millimetric mediastinal lymph nodes: SUVmax 3), indicating a positive response to treatment.

**Table 1:** Alpelisib impact in tumor markers evolution.

Initiation of Alpelisib	Ca 15.3 U/mL	CEA ng/dL
Before	169.1	18.01
4 weeks later	111.4	12.57
8 weeks later	87.7	5.48
13 weeks later	70.7	5.52

One month later the patient restarted alpelisib at a lower dose (250 mg/day). The onset of hyperglycemia was similar to baseline. The treatment course and the insulin dosage that were required to achieve an optimal metabolic control did not differ from the previously treatment regimen described. At 7-month follow-up, HbA1c was 8.1% and alpelisib tolerance was adequate, with no other adverse events attributable to the drug.

## Discussion

Hyperglycemia is the main adverse effect of alpelisib and the most frequent factor leading to discontinuation or dose decrease, being the cause of permanent withdrawal in 6.3% of the patients (3). Baseline pre-diabetes and diabetes, body mass index (BMI) ≥30 kg/m2 or age ≥75 years are risk factors for developing hyperglycemia. These risk factors were present in 74.7% of patients with any grade of hyperglycemia and in 86.2% of patients with grade 3 (160-250 mg) or 4 (>250 mg/dl) hyperglycemia (8). It is noteworthy that Clinical Trial SOLAR-1 Study protocol did not specifically include daily glycemic self-monitoring during the first weeks, thus the detection of hyperglycemia and the initiation of antidiabetic treatment may have been delayed in many cases. A better previous metabolic control decreases the occurrence and seriousness of alpelisib-induced hyperglycemia, as well as drug discontinuation and withdrawal rates (9). In our case report, blood glucose monitoring using FGM was conducted from the start of alpelisib treatment and leaded to and early face-to-face correction of hyperglycemia, in addition to telematic interventions using the corresponding web-based data management platform linked to our center.

The safety of alpelisib in patients with type 1 and uncontrolled type 2 diabetes has not been established as these patients have been excluded from clinical trials (6). Severe hyperglycemia, in some cases associated with hyperglycemic hyperosmolar nonketotic syndrome (HHNKS) or ketoacidosis, has been observed in patients treated with alpelisib, with fatal outcome in some cases of ketoacidosis (7). Glucose monitoring should be a must as early as treatment with alpelisib is introduced, especially in the first weeks, as hyperglycemia may occur with a rapid onset after starting treatment (8, 9).

Despite the lack of detailed information available on the pharmacokinetics of alpelisib, FGM has been helpful in understanding the pharmacodynamic aspects of alpelisib and provides valuable information that correlates with its pharmacokinetic properties. There are barely any studies describing in sufficient detail the day-to-day pattern related to alpelisib administration, the rapid onset alpelisib-induced hyperglycemia and its prompt reversibility (10). In this regard, the information provided by the FGM has been extremely helpful in understanding the pharmacodynamic aspects of alpelisib and has also provided valuable information that correlates with its pharmacokinetic properties. As reflected in summary of product characteristics, alpelisib has a short half-life, which, independent of dose and time, is 8 to 9 hours at steady state with 300 mg once daily, making the hyperglycemic effect nonexistent for at least 24 hours after the next dose administration, as it is shown in days 2 and 3 in **Figure 1**. Steady-state plasma levels of alpelisib after daily dosing can be expected to be reached on day 3 following onset of therapy in most patients. It is noteworthy that presentation of hyperglycemia is fast, already observed 24 hours after initiation of therapy. FGM shows severe and persistent hyperglycemia during most of the day, with a significant downward effect in the 4 hours after each daily intake, which evidences the strong but transitory effect of the drug enzyme blockade. This is in accordance with Alpelisib’s pharmacokinetic profile: after oral administration of alpelisib, median time to reach peak plasma concentration (Tmax) ranged between 2.0 to 4.0 hours, independent of the dose. This explains the improvement or normalization of glycemia in these 4 hours following administration (8). Our patient, who was taking alpelisib in lunchtime (main meal of the day -14.30h-), had to discontinue the corresponding insulin bolus with this meal due to the tendency towards spontaneous glycemic normalization in the first part of the afternoon and the risk of favoring hypoglycemia in this period. Nonetheless, hyperglycemia was increasing as the afternoon proceeded and rapid-acting insulin boluses were needed for afternoon snack and dinner. We believe that the observed afternoon glucose nadir in AGP, which precedes the severe hyperglycemia in the evening is due to the short half-life of alpelisib and a certain delay in the enzyme inhibition after its administration, despite we have not found pharmacokinetic studies supporting this behavior. In addition, C-peptide level is remarkable in accordance with drug pharmacodynamics, consistent with a significant insulin resistance. The majority of insulin’s actions begin with activation of the phosphatidylinositol-3-kinase (PI3K)/AKT signaling pathway, which plays a central role in the activation of glucose transport, mainly increasing glucose uptake and glycogen deposition in liver and skeletal muscles. This PI3K/AKT insulin signaling pathway triggers when insulin binds to its receptor. This binding triggers kinase activity, which leads to phosphorylation of the receptor and activation of PI3K, which in turn activates AKT, resulting in the translocation of the GLUT4 glucose transporter from an intracellular pool to the plasma membrane in insulin target organs. This results in glucose uptake to the inside of the cells while reducing blood glucose level. Consequently, any impairment of PI3K/AKT pathway will result in insulin resistance and in hyperglycemia (11, 12). Alpelisib PI3K inhibitor therapy causes a disturbance in the signaling pathway, blocking glucose uptake by skeletal muscle and adipose tissue and activating hepatic glyconeogenesis (6). On the basis of alpelisib mechanism of action and with the aim of slowing down this strong insulin resistance and hyperglycemia, it is worth mentioning that insulin sensitizers as metformin and pioglitizone may be preferred to insulin secretagogues to manage hyperglycemia (6), and concomitant insulin treatment may be necessary (8). More data are needed to support the use of other antidiabetic medication as sodium glucose cotransporter 2 inhibitors. Patients with a history of diabetes mellitus may require intensified diabetic treatment. Glucose monitoring should always be performed in patients treated with alpelisib, especially in patients with diabetes and prediabetes. Compared to self-monitoring of capillary blood glucose, continuous glucose monitoring system provides a close monitoring of glycemic behavior in a non-invasive way, in order to early detect and treat hyperglycemia. Any delay can lead to a worsening in metabolic control resulting in the discontinuation or reduction of alpelisib. Despite the influence on survival rate of alpelisib-induced hyperglycemia is currently unclear, the effect of alpelisib is dose-dependent. The higher the dose, the greater the efficacy in terms of time to disease progression (6). Any interruption or discontinuation of alpelisib which would lead to a decrease in its effectiveness, and consequently would deny patients an effective treatment with an impact on quality of life and survival.

## Conclusion

We report the case of a severe hyperglycemia associated with alpelisib treatment in a patient with metastatic breast cancer, which highlights PI3K inhibitor pharmacodynamic aspects, its influence in glycemic control and the required treatment approach. Glucose monitoring should always be performed in patients treated with alpelisib, especially in patients with diabetes and prediabetes. It is crucial to anticipate in these patients. Any delay can lead to a worsening in metabolic control resulting in the discontinuation or reduction of alpelisib, which would lead to a decrease in its effectiveness, and consequently would deny patients an effective treatment with an impact on survival.

## Data Availability Statement

The original contributions presented in the study are included in the article/ Supplementary Material. Further inquiries can be directed to the corresponding author.

## Ethics Statement

Written informed consent was obtained from the individual(s) for the publication of any potentially identifiable images or data included in this article.

## Author Contributions

All authors listed in the manuscript have contributed to the work: BP and AA contributed to the preparation of the manuscript. FS, AA, and AB contributed to the patient’s clinical care. AA and MM contributed to revision and final approval of the manuscript.

## Conflict of Interest

The authors declare that the research was conducted in the absence of any commercial or financial relationships that could be construed as a potential conflict of interest.

## Publisher’s Note

All claims expressed in this article are solely those of the authors and do not necessarily represent those of their affiliated organizations, or those of the publisher, the editors and the reviewers. Any product that may be evaluated in this article, or claim that may be made by its manufacturer, is not guaranteed or endorsed by the publisher.
